# The Use of Copper Amalgam

**Published:** 1888-10

**Authors:** Sidney S. Stowell

**Affiliations:** Pittsfield, Mass.


### THE USE OF COPPER AMALGAM.

BY SIDNEY S. STOWELL, D.D.S., OF PITTSFIELD, MASS.

Read before the Union Meeting of the Connecticut Valley and Massachusetts Dental Societies, Boston, Mass., July 10, 1888.

The subject named has been so thoroughly canvassed that I will not undertake to give its origin or history. It is my purpose at this time to try and bring before the profession some important points with regard to the manipulation of this most valuable tooth saving material, which has been called by no less a man than Fletcher, an absolutely permanent filling for cavities in the teeth; this I claim cannot so truly be said of any other material used for that purpose; it is without doubt the oldest plastic filling material known, it having been used by the English for more than sixty years. One make, known as Sullivans, having been made after the same formula for that length of time.

The saving qualities of copper amalgam need no argument to prove them second to no filling material known to the profession. It has stood the test of sixty years, and is still in its place. That it turns black is known to all. This is its only fault, and how small is it as compared with the great advantages which it possesses as a preserver of teeth. It *will not shrink*, a quality which in my judgment is not possessed to so great an extent by any other amalgam known. The chemical action which it exerts upon the tooth structure with which it comes in contact is, as a germicide, such that, when reasonable care has been used in its insertion, caries of the tooth at that point *cannot recur*. Think for one moment, gentlemen, of the force of this assertion, which, if it be true, the same cannot be said of any other filling material. I have studied well the manufacture and use of copper amalgam, as a filling material, for several years and with the most satisfactory results. I have also used many of the common high and low grade amalgams, manipulating them to the best of my ability, the rubber dam being used, and all care and skill exercised upon teeth of seemingly fair quality, but which in a year's time have decayed rapidly around my high grade amalgam filling.

My gold fillings in the incisors of the same mouth have stood well, but the cavities have become large in the proximal surface of the molars and in the wisdom teeth which were soft.

I have used copper amalgam in the same teeth without a single failure, although where high grade amalgam was used in one side of several of the teeth it failed as before. A record having been kept of these cases, and subsequent examinations made, have proved that copper amalgam has saved the teeth, where high grade amalgam has failed.

Teeth filled with copper amalgam do not discolor any more than where other amalgams are used, because there is no shrinkage, consequently no leaking, and, therefore, no discoloration.

The surface of the filling is somewhat darker than that of other amalgams; but the teeth are saved, and to all appearances for the life time of the patient. Had not you rather see in the mouth of your patient a filling, the surface of which is dark, and the tooth saved in a healthy condition, than a white filling, with a return of caries? In your own mouth which would you rather have? We fill teeth to save them; that is what our patients want; that is what they pay for.

Let us now consider the manipulation of copper amalgam which, as is generally known, is supplied to the profession by the trade, most of it being of English make. I have manufactured copper amalgam for some time, and do not consider that the English amalgam possess any superiority over some American makes.

As we receive the amalgam from the trade, it is put up in the form of bars or pellets, which are very hard; amalgamation having taken place, an excess of mercury being used, and the mass allowed to harden. A portion is placed in an iron spoon, and heated over an alcohol lamp or Bunsen burner until the mercury begins to ooze out. Now heat gently until it can be crushed by slight pressure; do not heat beyond that point. Triturate in a small glass mortar with pestle, and press out the excess of mercury through chamois' skin with pliers. My copper amalgam C. P., when treated in this way, will become quite hard in from fifteen to thirty minutes, and fully hard in one hour's time. If, however, it is heated beyond the point mentioned, it will be slow setting.

Thus a quick or slow setting amalgam may be obtained from the same beginning through difference in manipulation. By care and experience, the operator can soon acquire the ability to produce a quick or slow setting amalgam at will.

A quick setting amalgam may also be obtained by using as soon as the copper is combined with the mercury for the first time, or the mass has been allowed to harden ; but on account of the great inconvenience of making from the beginning a new lot of amalgam for each operation, the softening by heat of that already prepared is preferable.

Copper amalgam fillings may be topped off with any high grade or so-called " white alloy," or with gold, thereby rendering the operation more sightly without interfering with the saving qualities of the copper.

Amalgams in all their forms have been classed together and called " the lazy man's fillings."

I am not an amalgamist, nor a lazy man. I am a contour-with-gold-compound-proximal-bicuspid-and-molar man ; although I am happy to know of so valuable an adjunct to our usual methods of practice, whereby we may save a large class of teeth which, in my judgment, could not so permanently be done by any other means.

